# HLA-B^*^57 Allele Is Associated with Concomitant Anti-tuberculosis and Antiretroviral Drugs Induced Liver Toxicity in Ethiopians

**DOI:** 10.3389/fphar.2017.00090

**Published:** 2017-02-27

**Authors:** Zelalem Petros, Junko Kishikawa, Eyasu Makonnen, Getnet Yimer, Abiy Habtewold, Eleni Aklillu

**Affiliations:** ^1^Department of Pharmacology, School of Medicine, College of Health Sciences, Addis Ababa UniversityAddis Ababa, Ethiopia; ^2^Department of Laboratory Medicine, Karolinska Institutet-Karolinska University HospitalStockholm, Sweden

**Keywords:** antiretroviral drugs, anti-tuberculosis, drug induced hepatotoxicity, DILI, Ethiopian, HIV, HLA, HLA-B^*^57

## Abstract

Drug-induced liver injury (DILI) is a known adverse effect of both anti-tuberculosis (anti-TB) and antiretroviral (ARV) drugs. Recent studies highlight the implications of genetic predispositions to DILI. We performed a case-control study to identify Human Leukocyte Antigen-B (HLA-B) variant alleles associated with anti-TB and ARV co-treatment induced liver toxicity in Ethiopian TB and HIV co-infected patients. A total of 495 newly diagnosed TB and HIV co-infected patients were enrolled and received rifampicin based anti-TB and efavirenz based ARV therapy. Change in liver enzyme level from baseline was monitored 1st, 2nd, 4th, 8th, 12th, and 24th weeks after treatment initiation to identify patients who developed DILI (cases) and those who did not (treatment tolerants). Genomic DNA from 46 cases and 46 sex and age matched treatment tolerants were genotyped for HLA-B variant alleles using Olerup SSP®HLA-B DNA Typing Kits. The proportion of HLA-B^*^57 allele carriers in DILI cases (37.0%), particularly in those who developed cholestatic type of DILI (44.8%) was significantly higher compared with those who tolerated the treatment (2.2%). The HLA-B^*^57 allele frequency was significantly higher in cases (25%) than treatment tolerants (1.1%). In a multivariate logistic analysis, the proportion of patients carrying HLA-B^*^57 (*P* = 0.002) and HLA-B^*^14 (*P* = 0.014) alleles were significantly higher in DILI cases compared with treatment tolerants. HLA-B^*^57 was significantly associated with cholestatic (*P* = 0.001) and mixed (*P* = 0.017) types of liver toxicity, and mild-to-moderate severity (*P* = 0.001). Of all HLA-B^*^57 alleles detected, HLA-B^*^57:03 accounted 58.3% and HLA-B^*^57:02 accounted 41.7%. HLA-B^*^57:01 was not detected. The variant allele frequencies of HLA-B^*^57:03 (15.2 vs. 0%) and HLA-B^*^57:02 (9.8 vs. 1.1%) were significantly higher in the DILI cases than treatment tolerants (*P* < 0.03). We conclude that HLA-B^*^57 alleles (B^*^57:03 and B^*^57:02) confer susceptibility to the development of anti-TB and ARV drugs co-treatment induced liver toxicity, which is mainly of cholestatic type. The possible association of HLA-B^*^14 with anti-TB and ARV drugs co-treatment induced liver toxicity requires further investigations.

## Introduction

Tuberculosis (TB) is the most common opportunistic infection associated with human immunodeficiency virus (HIV) infection, and co-treatment of the two diseases is recommended (Harries et al., 2009). However, simultaneous treatment of TB and HIV infections is challenging due to drug interactions and overlapping toxicities (Cohen and Meintjes, 2010). Antiretroviral (ARV) and anti-tuberculosis (anti-TB) Drugs-induced liver injury (DILI) is a common adverse event, which can be fatal if therapy is not interrupted or changed on time (Devarbhavi et al., 2013; Naidoo et al., 2015; Shamanna et al., 2016). TB-HIV co-infected patients on anti-TB and ARV co-therapy are at a higher risk of developing DILI than TB or HIV only infected patients receiving monotherapy (Yimer et al., 2011, 2014; Mugusi et al., 2012). A recent study in TB/HIV patients on anti-TB and antiretroviral therapy (ART) with high levels of immune activation demonstrated impaired isoniazid clearance, implicating the need for exploring immune response and the risk of DILI (Vinnard et al., 2016). Up to 32% of HIV patients on ART discontinue their treatment or switch therapy mainly due to DILI (Bica et al., 2001), and genetic predisposition contributes partly (Lubomirov et al., 2011). Treatment interruption may increase the risk for developing of multidrug-resistant TB (MDR-TB) and HIV/AIDS (Hirpa et al., 2013). Thus, identifying genetic markers for drug-induced liver toxicity is valuable to identify high-risk patients and to introduce appropriate measures.

Both HIV and TB remain a major problem and co-infection is common in most Sub-Saharan African (SSA) countries including Ethiopia, the second most densely populated country in Africa with an estimated population size of 100 million. Ethiopia is listed among the top 20 high-TB burden countries globally, and one of the high MDR-TB burden countries (Biadglegne et al., 2014; World-Health-Organization, 2016). The rate of new HIV infection in Ethiopia is declining with the estimated number of people living with HIV being 769, 600 in 2014 (World-Health-Organization^1^). The scale of ART is increasing in the country as part achieving the UNAID/WHO “90-90-90” target: to diagnose 90% of all HIV positive people, provide ART for 90% of those diagnosed and achieve viral suppression for 90% of those treated, by 2020 (UNAIDS, 2014). ART and anti-TB drug-induced liver toxicity is a common problem in Ethiopia causing treatment discontinuation and hence MDR-TB (Hirpa et al., 2013; Yimer et al., 2014).

Genetic variations in HLA gene is implicated with susceptibility to T-cell mediated adverse events to a wide range of pharmaceuticals making it a candidate gene relevant to pharmacogenetic studies (Barbarino et al., 2015). HLA alleles that are reported to be association with increased risk of idiosyncratic DILI include: HLA-DQB1^*^02:01 and DQB1^*^05 to anti-TB drugs (Sharma et al., 2002; Chen et al., 2015), and HLA-B^*^58:01 and DRB1^*^01:02 to nevirapine-containing ARV regimens (Phillips et al., 2013). HLA-B^*^57:01 and A^*^33:03 variant alleles were also reported as genetic markers for idiosyncratic liver injury induced by flucloxacillin (Daly et al., 2009) and ticlopidine (Hirata et al., 2008) respectively. Susceptibility to amoxicillin-clavulanate-induced liver injury is influenced by multiple HLA class I and II alleles (Lucena et al., 2011). A large genome-wide association study found a strong association of amoxicillin-clavulanate induced liver injury with HLA-A^*^02:01, HLA-DQB1^*^06:02, and DRB1^*^15:01 variant alleles (Lucena et al., 2011). Genetic screening for HLA-B^*^57:01 and subsequent treatment modifications have been shown to reduce incidence of life-threatening hypersensitivity to abacavir in HIV/AIDS patients carrying the allele (Hughes et al., 2008; Mallal et al., 2008) and HLA-B^*^15:02 to carbamazepine in Southeast Asian carriers (Amstutz et al., 2014).

Most previous reports investigating genetic risk factors for anti-TB and ARV drugs-induced liver toxicity focused on drug metabolizing enzymes and transporter proteins (Lee et al., 2010; Yimer et al., 2011, 2012). Previously we reported the association of high efavirenz plasma concentration and CYP2B6^*^6 genotype with DILI in TB-HIV patients (Yimer et al., 2011, 2012; Mugusi et al., 2012). However, only a few studies have explored the association of HLA genes with anti-TB or ARV drugs-induced liver toxicity. Therefore, in this study, we aimed to investigate the possible associations between HLA-B alleles, and anti-TB and ARV drugs co-treatment induced liver injury in TB and HIV co-infected patients in Ethiopia.

## Methods

### Study design and participants

Using a case-control comparative study design, we analyzed data from newly diagnosed TB and HIV co-infected patients, who were enrolled and followed up prospectively to identify the incidence, the pattern, and severity of anti-TB and ARV drugs-induced liver toxicity in Ethiopian patients (Yimer et al., 2014). In brief, 495 TB and HIV co-infected patients with CD4 count ≤ 200 cells/mm^3^ were recruited from three health institutions: Kazanchis and Beletshachew health centers, and Tikur Anbessa Specialized Hospital in Addis Ababa, Ethiopia, from June 2007 to June 2012. The inclusion criteria were TB and HIV co-infected men and non-pregnant women with age 18 years old and above. Patients were excluded if they had a history of prior treatment for TB/HIV or known pre-existing liver disease.

The study protocol was approved by the Institutional Review Board of College of Health Sciences, Addis Ababa University, the National Research Ethics Review Committee of Ethiopia, and Ethical Review Board of Karolinska Institutet, Sweden. Written informed consent was obtained from all the study participants in accordance with the Declaration of Helsinki.

### Drug treatment

All the study participants received first line ARV drugs containing efavirenz and lamivudine with tenofovir, zidovudine, or stavudine. A short-course anti-TB regimen consisting of rifampicin, isoniazid, pyrazinamide, and ethambutol for the first 2 months followed by rifampicin and isoniazid for the next 4 months was given. The patients did not receive other known hepatotoxic drugs concurrently, except co-trimoxazole prophylaxis that was given for TB and HIV co-infected patients according to the National Treatment Guideline. Change in liver enzymes levels from baseline was monitored on the 1st, 2nd, 4th, 8th, 12th, and 24th weeks after initiation of treatment.

### Case definitions, severity grade, and pattern of liver toxicity

The criteria set by the International DILI Expert Working Group were used for DILI case definitions and pattern of liver injury determination (Aithal et al., 2011). The upper limit of normal (ULN) for liver enzymes used for the study population were alanine aminotransferase (ALT 33 U/L for male; 29 U/L for female), aspartate aminotransferase (AST, 41 U/L), alkaline phosphatase (ALP, 128 U/L), and 1.0 mg/dL for total bilirubin (Yimer et al., 2014). All cases recruited met at least one of the following criteria: (1) ALT ≥ 5xULN, (2) ALP ≥ 2xULN, or (3) ALT ≥ 3xULN along with total bilirubin (T Bil) ≥ 2xULN. Treatment tolerants (controls) were individuals who were on anti-TB and ARV drugs co-treatment but did not fulfill the case definitions for DILI and had not presented with clinical signs and symptoms consistent with DILI in the follow-up period.

The pattern of liver toxicity was defined using *R*-value, where *R* = (ALT/ULN)/(ALP/ULN). Cases were categorized as hepatocellular (*R* ≥ 5), cholestatic (*R* ≤ 2), or mixed (2 < *R* < 5) pattern of DILI. Clinical severity grading was determined by employing the highest measured values for each of the biochemical parameters (Yimer et al., 2014). Patients with grades one and two severities were grouped together into a “mild-to-moderate” group and those with grades three and four into a “severe” group. Causality assessment for DILI was performed using Roussel Uclaf Causality Assessment Method (RUCAM; Danan and Benichou, 1993).

Among the 495 TB and HIV co-infected patients involved in the initial cohort, 120 experienced DILI in the follow-up period (Yimer et al., 2014). Of these, 80 cases and 275 treatment tolerants had adequate DNA available for further analysis. After excluding patients that had abnormal liver biochemistry prior to starting treatment, or patients who had serological test positive for either hepatitis B virus surface antigen or anti-hepatitis C virus antibody, 46 cases and 46 treatment tolerants that have complete clinical data and matched with respect to gender and age in a 1:1 ratio were used for the current study.

### HLA-B genotyping

Genomic DNA was isolated from peripheral blood using QIAamp DNA Maxi Kit (QIAGEN GmbH. Hilden. Germany). We first screened for HLA-B variant alleles using a low-resolution (two digits) genotyping. HLA-B genotyping was performed using low-resolution Olerup SSP®HLA-B Typing Kit (Olerup SSP AB, Franzengatan 5, SE-112 51 Stockholm, Sweden). Allele-specific polymerase chain reaction (PCR), using sequence-specific primers was done according to the protocol and recommendations of the manufacturer. The amplified PCR products were analyzed using 2% agarose gel, and the HLA-B allele types were determined using HELMBERG-SCORE software. Low-resolution typing results were recorded with the 2-digit code to ensure a uniform level of HLA resolution for the alleles.

As a next step, high resolution (four digits) typing were done for HLA-B variant alleles that showed significant association with DILI based on the low-resolution genotyping data. HLA-B^*^57 exhibited a significant association with DILI, and high-resolution subtyping was performed for all HLA-B^*^57 allele carriers using Olerup SSP®HLA-B^*^57:01 Typing Kit (Olerup SSP AB, Franzengatan5, SE-112 51 Stockholm, Sweden) according to the protocol and recommendations of the manufacturer.

### Statistical analysis

Continuous variables were presented by a mean and standard deviation, and categorical variables as numbers and percentages. Univariate logistic regression analysis was used to identify potential independent risk factors for anti-TB and ARV drugs co-treatment induced liver toxicity. Variables with *P* < 0.1 in the univariate analysis were included in a multivariate logistic analysis. The strength of the associations was estimated by calculating the odds ratio (OR) and 95% confidence interval (CI). Fisher's exact test was used for HLA-B alleles with <5 expected cell count in a 2 × 2 table. To reduce bias in estimating the OR, Haldane's modification was employed (Haldane, 1956) i.e., whenever a zero-count cell was encountered, 0.5 was added to all cells in the 2 × 2 table. *P* < 0.05 were considered statistically significant. The corrected *P*-values (*Pc*) were adjusted by using Bonferroni's correction for multiple comparisons (18 tests) to account for the number of HLA-B alleles observed in the study participants. The statistical analysis was performed using IBM SPSS Statistics for Windows, Version 20.0 (IBM Corp., Armonk, NY, USA).

## Results

A total of 92 TB and HIV co-infected patients on anti-TB and ARV drugs co-treatment were involved in this study; 46 treatment induced liver toxicity cases and 46 sex and age matched treatment tolerants. The demographics and clinical characteristics of the study participants are described in Table 1.

**Table 1:** **Demographics and clinical characteristics of the study participants**.

**Characteristics**	**Cases**	**Treatment tolerants**	**Univariate analysis**		**Multivariate analysis**	
	**(*N* = 46)**	**(*N* = 46)**	***P***	**OR (95% CI)**	***P***	**OR (95% CI)**
Sex (M/F)	22/24	22/24	1.00	1.00 (0.44–2.27)		
Age (yrs), mean (*SD*)	35.4 (9.3)	35.3 (8.7)	0.95	0.99 (0.95–1.05)		
BMI (kg/m^2^), mean (*SD*)	18.4 (2.4)	19.1 (3.5)	0.25	1.09 (0.94–1.26)		
CD4 count (per mm^3^), mean (*SD*)	69.3 (48.7)	94.2 (51.4)	**0.02**	1.01 (1.01–1.02)	**0.03**	1.01 (1.00-1.02)
Viral load (copies/ml), log mean (*SD*)	5.3 (1.0)	5.0 (1.0)	0.20	0.72 (0.44–1.19)		
Karnofsky score	82.8 (13.6)	88.7 (13.4)	**0.04**	1.03 (1.00–1.07)	0.06	1.03 (0.99–1.07)
**LFT VALUES, MEAN (*****SD*****)**						
Baseline ALT (U/L)	25.5 (9.8)	27.8 (8.3)	0.22	1.03 (0.98–1.08)		
Baseline AST (U/L)	32.7 (10.3)	31.9 (9.6)	0.70	0.99 (0.95–1.03)		
Baseline ALP (U/L)	99.5 (19.3)	104.3 (19.3)	0.23	1.01 (0.99–1.04)		
Baseline TBil (mg/dL)	0.7 (0.5)	0.7 (0.4)	0.71	0.84 (0.34–2.10)		
**ARV REGIMEN**, ***N*** **(%)**						
TDF/3TC/EFV	15 (32.6)	12 (26.1)	0.49	1.37 (0.56–3.38)		
ZDV/3TC/EFV	16 (34.8)	16 (34.8)	1.00	1.00 (0.42–2.36)		
D4T/3TC/EFV	15 (32.6)	18 (39.1)	0.52	0.75 (0.32–1.78)		
**PATTERN OF LIVER INJURY, ***N*** (%)**						
Cholestatic	29 (63.0)					
Hepatocellular	2 (4.4)					
Mixed	15 (32.6)					
**SEVERITY GRADE, ***N*** (%)**						
Mild-to-moderate	39 (84.8)					
Severe	7 (15.2)					
**RUCAM SCALE, ***N*** (%)**						
Definite (score > 8)	30 (65.2)					
Probable (score 6–8)	12 (26.1)					
Possible (score 3–5)	4 (8.7)					

*3TC, Lamivudine; ALP, Alkaline phosphatase; ALT, Alanine aminotransferase; ARV, Antiretroviral; AST, Aspartate aminotransferase; BMI, Body mass index; CI, Confidence Interval; D4T, Stavudine; EFV, Efavirenz; HIV, Human immunodeficiency virus; LFT, Liver function test; OR, Odds ratio; RUCAM, Roussel Uclaf Causality Assessment Method; SD, Standard deviation; T Bil, Total bilirubin; TB, Tuberculosis; TDF, Tenofovir; ZDV, Zidovudine. P-value < 0.05 is highlighted in bold*.

In the univariate analysis, there were statistically significant differences in the CD4 count and Karnofsky score between DILI cases and treatment tolerants (*P* < 0.05). In a multivariate logistic analysis, baseline CD4 count remained as significant predictors of anti-TB and ARV drugs co-treatment induced liver injury. There were no statistically significant differences in the baseline liver enzyme levels and type of ARV regimens used between the DILI cases and treatment tolerants. More than half of the DILI cases developed the cholestatic type of liver toxicity, and 85% of the cases had mild-to-moderate severity of liver toxicity. All of the cases had a minimum score of three (“possible”) in RUCAM scoring system for DILI.

*HLA-B* genotype result from the low resolution typing for each study participant is presented in Supplementary Table 1. Comparison of *HLA-B* allele carriers' proportions between patients who developed DILI vs. treatment tolerants is presented in Table 2. A total of 18 *HLA-B* variant alleles were detected (Table 2). In the univariate analysis, the proportion of HLA-B^*^57 and HLA-B^*^14 alleles carriers were significantly higher in DILI cases than treatment tolerants. Association of being a carrier of HLA-B^*^57 with increased risk for DILI remained significant after correction for multiple testing. On the other hand, HLA-B^*^41 was negatively associated with DILI. The multivariate logistic analysis retained HLA-B^*^57 and HLA-B^*^14 as significant predictors of concomitant anti-TB and ARV drugs induced liver injury. For HLA-B^*^57, the association maintained after correcting for multiple comparisons (*Pc* = 0.036).

**Table 2:** **Comparison of proportion of ***HLA-B*** allele carriers between patients who developed ARV and anti-tuberculosis drugs induced liver injury (cases) and patients who did not (treatment tolerants)**.

**HLA-B alleles**	**DILI cases (*N* = 46)**	**Treatment tolerants (*N* = 46)**	**Univariate analysis**			**Multivariate analysis**	
	***N*** **(%)**	***N*** **(%)**	***P***	***Pc***	**OR (95% CI)**	***P***	**OR (95% CI)**
B^*^57	17 (37.0%)	1 (2.2%)	**0.002**	**0.036**	26.38 (3.33–209.07)	**0.002**	30.08 (3.44–263.11)
B^*^14	10 (21.7%)	3 (6.5%)	**0.047**	0.845	3.98 (1.02–15.58)	**0.014**	7.51 (1.50–37.68)
B^*^41	3 (6.5%)	10 (21.7%)	**0.047**	0.845	0.25 (0.06–0.98)	0.112	0.26 (0.05–1.37)
B^*^15	8 (17.4%)	16 (34.8%)	0.06	1.000	0.40 (0.15–1.05)	0.526	0.68 (0.21–2.23)
B^*^08	0	4 (8.7%)	0.12		1.48 (0.01–1.94)^*^		
B^*^18	0	3 (6.5%)	0.24		0.13 (0.01–2.66)^*^		
B^*^44	2 (4.3%)	5 (10.9%)	0.25		0.37 (0.07–2.03)		
B^*^13	5 (10.9%)	8 (17.4%)	0.37		0.58 (0.17–1.93)		
B^*^58	4 (8.7%)	2 (4.3%)	0.41		2.10 (0.36–12.05)		
B^*^51	6 (13.0%)	4 (8.7%)	0.50		1.58 (0.41–6.00)		
B^*^53	4 (8.7%)	3 (6.5%)	0.70		1.37 (0.29–6.47)		
B^*^07	10 (21.7%)	9 (19.6%)	0.80		1.14 (0.42–3.14)		
B^*^49	10 (21.7%)	10 (21.7%)	1.00		1.00 (0.37–2.69)		
B^*^35	1 (2.2%)	0	1.00		3.07 (0.12–77.25)^*^		
B^*^37, ^*^39, ^*^50, ^*^73	0	2 (4.3%)	0.50		0.19 (0.01–4.10)^*^		

* *Haldane's modification*.

*ARV, Antiretroviral; CI, Confidence Interval; HLA, Human Leukocyte Antigen; OR, Odds ratio; TB, Tuberculosis*.

*P, P-values were calculated by Fisher's exact test comparing the positive alleles in cases with those of treatment tolerants*.

*Pc, Corrected P-values were adjusted by using Bonferroni's correction for multiple comparisons to account for the observed 18 HLA-B alleles. P-value < 0.05 is highlighted in bold*.

Associations between HLA-B^*^57, HLA-B^*^14, and HLA-B^*^41 with the pattern and severity of liver toxicity was evaluated by comparing the proportion of allele carriers between DILI cases and treatment tolerants (Table 3). Compared with treatment tolerants, the proportion of HLA-B^*^57 allele carriers were significantly higher in cholestatic and mixed types of DILI. In addition, the proportion of HLA-B^*^14 allele carriers were higher in the mixed type of DILI cases than the treatment tolerants. There were no statistically significant differences in the hepatocellular type of DILI. Compared with treatment tolerants, the proportion of patients carrying HLA-B^*^57 allele was significantly higher in the mild-to-moderate DILI group, and those carrying HLA-B^*^14 in the severe DILI group were over-represented. There was a statistically significant difference in the proportion of HLA-B^*^41 allele carriers in the mild-to-moderate DILI group compared with treatment tolerants.

**Table 3:** **Association of HLA-B alleles with the pattern and severity of drug-induced liver injury**.

**Characteristics**	HLA-B^*^57 **carriers**			HLA-B^*^14 **carriers**			HLA-B^*^41 **carriers**		
	***N*** **(%)**	***P***	**OR (95% CI)**	***N*** **(%)**	***P***	**OR (95% CI)**	***N*** **(%)**	***P***	**OR (95% CI)**
**DILI PATTERN**									
Treatment tolerants (*n* = 46)	1 (2.2)	–	–	3 (6.5)	–	–	10 (21.7)	–	–
Cholestatic (*n* = 29)	13 (44.8)	**0.001**	36.6 (4.4–302.3)	5 (17.2)	0.17	3.0 (0.7–13.6)	2 (6.9)	0.11	0.3 (0.1–1.3)
Hepatocellular (*n* = 2)	0	1.00	6.1 (0.2–190.1)^*^	1 (50.0)	0.16	14.3(0.7–29.4)	0	1.00	0.7 (0.1–15.6)^*^
Mixed (*n* = 15)	4 (26.7)	**0.017**	16.4 (1.7–161.3)	4 (26.7)	**0.048**	5.2 (1.0–26.8)	1 (6.7)	0.22	0.3 (0.1–2.2)
**SEVERITY GRADE**									
Mild-to-moderate (*n* = 39)	16 (41.0)	**0.001**	31.3 (3.9–251.0)	7 (17.9)	0.12	3.1(0.8–13.1)	2 (5.1)	**0.04**	0.2 (0.1–1.0)
Severe (*n* = 7)	1 (14.3)	0.173	2.7 (0.6–11.7)	3 (42.9)	**0.01**	10.8 (1.6–71.9)	1 (14.3)	0.65	0.6 (0.1–5.6)

* *Haldane's modification*.

*CI, Confidence Interval; HLA, Human Leukocyte Antigen; OR, Odds ratio. P-value < 0.05 is highlighted in bold*.

*HLA-B* variant alleles that showed significant association with DILI from the low-resolution genotyping were further subjected to high resolution (four digit) testing to identify the sub-variant allele. The result from high resolution genotyping was interpreted in conjunction with the results from the prior low-resolution typing. HLA-B^*^57 was the only variant allele that exhibited a significant association with DILI after correcting for multiple testing. Thus, high-resolution typing was done for all subjects who were genotyped as carriers of HLA-B^*^57 in the low-resolution typing (see Table 4).

**Table 4:** High resolution genotyping data for all HLA-B^*^57 allele carriers stratified by DILI type.

**Patient No**.	**Age**	**Sex**	**Status**	**DILI type**	**Allele 1**	**Allele 2**
1	25	F	Case	Cholestatic	B^*^57:03	B^*^57:03
2	34	F	Case	Cholestatic	B^*^57:03	B^*^57:03
3	45	M	Case	Cholestatic	B^*^57:03	B^*^57:03
4	25	M	Case	Cholestatic	B^*^57:02	B^*^57:02
5	28	F	Case	Cholestatic	B^*^57:03	B^*^58:01
6	37	F	Case	Cholestatic	B^*^57:02	B^*^51:08
7	28	F	Case	Cholestatic	B^*^57:03	B^*^49:01
8	55	M	Case	Cholestatic	B^*^57:02	B^*^39:12
9	45	F	Case	Cholestatic	B^*^57:02	B^*^58:01
10	30	F	Case	Cholestatic	B^*^57:03	B^*^53:01
11	60	M	Case	Cholestatic	B^*^57:03	B^*^13:02
12	30	F	Case	Cholestatic	B^*^57:03	B^*^53:01
13	30	F	Case	Cholestatic	B^*^57:02	B^*^44:02
14	30	F	Case	Mixed	B^*^57:03	B^*^57:03
15	31	M	Case	Mixed	B^*^57:02	B^*^57:02
16	49	M	Case	Mixed	B^*^57:03	B^*^58:01
17	38	F	Case	Mixed	B^*^57:02	B^*^49:01
18	32	F	Control	Treatment tolerant	B^*^57:02	B^*^41:02

DILI, Drug induced liver injury. B^*^57:02 and B^*^57:03 alleles are highlighted in bold.

Of all HLA-B^*^57 alleles identified, HLA-B^*^57:03 accounted 58.3% and HLA-B^*^57:02 accounted 41.7%. HLA-B^*^57:01 was not detected. Of the HLA-B^*^57 allele carrier cases, 10 (58.8%) had HLA-B^*^57:03 (four homozygous) and the rest seven (41.2%) had HLA-B^*^57:02 (two homozygous). There was only one HLA-B^*^57 allele carrier (heterozygous for HLA-B^*^57:02) out of the 46 treatment-tolerants. The proportion of HLA-B^*^57:03 and B^*^57:02 allele carriers were significantly higher in the DILI cases than treatment tolerants [*P* = 0.01, OR = 26.8 (1.5–47.2) and *P* = 0.03, OR = 8.1 (1.0–68.6)], respectively.

Comparisons of HLA-B^*^57, ^*^14, and ^*^41 (from the low-resolution genotyping) as well as HLA-B^*^57:02 and B^*^57:03 (from the high-resolution genotyping) allele frequencies between DILI cases and treatment tolerants is presented in Table 5. The overall allele frequency of HLA-B^*^57 was higher (13.0%) than that of the other *HLA-B* alleles (7.6% for B^*^14 and 7.1% for B^*^41). The allele frequency of HLA-B^*^57 was higher in DILI cases (25.0%) compared to treatment tolerants (1.1%). The HLA-B^*^57:03 and B^*^57:02 allele frequencies in DILI cases (15.2 and 9.8%) were higher than the treatment tolerants (0 and 1.1%), respectively.

**Table 5:** **Comparison of ***HLA-B*** allele frequencies distribution between patients who developed ARV and anti-tuberculosis drugs induced liver toxicity (cases) and patients who did not (treatment tolerants)**.

**HLA-B allele**	**DILI cases (*****N*** = **46)**		**Treatment tolerants (*****N*** = **46)**		***P*****-value**	**Odds ratio**	
	**Observed frequency**	**95% CI**	**Observed frequency**	**95% CI**		**OR**	**95% CI**
HLA-B^*^57	0.250	0.162–0.338	0.011	0.004–0.032	<0.001	30.33	4.00–230.3
HLA-B^*^14	0.130	0.062–0.199	0.033	0.004–0.069	0.06	3.30	1.02–10.65
HLA-B^*^41	0.033	0.004–9.069	0.109	0.045–0.172	0.08	0.28	0.07–1.04
HLA-B^*^57:03	0.152	0.079–0.226	0	0			
HLA-B^*^57:02	0.098	0.037–0.159	0.011	0.001–0.032			

*OR, Odds Ratio; CI, Confidence interval; HLA, Human leukocyte antigen*.

## Discussion

In the present study, we investigated the association of *HLA-B* variant alleles with risk for concomitant anti-TB and ARV drugs induced liver toxicity. The proportion of HLA-B^*^57 allele carriers in Ethiopian patients who developed anti-TB and ARV drugs induced liver toxicity (37.0%), particularly in those who developed cholestatic type of liver toxicity (44.8%) was significantly higher compared with those who tolerated the treatment (2.2%). The proportion of HLA-B^*^14 allele carriers who developed DILI (21.7%) was also significantly higher compared with the treatment tolerants (6.5%). These indicate that HLA-B^*^57 and B^*^14 allele carriers might be at a higher risk of developing anti-TB and ARV drugs co-treatment induced liver toxicity. Accordingly, these variant alleles might play important roles in the pathogenesis of immune-mediated liver toxicity during anti-TB and ARV drugs co-treatment. The HLA-B^*^57 and B^*^14 molecules may function as endogenous antigen presenting molecules for the drugs/metabolites to HLA-restricted cytotoxic T-cell activation (Pichler, 2002). To our knowledge, this is the first report to investigate the association of HLA-B^*^57, B^*^14, and B^*^41 alleles with anti-TB and ARV drugs co-treatment induced liver toxicity.

The HLA-B^*^57 variant allele, which was observed in significantly higher proportion among DILI cases than treatment tolerants, had a high specificity (97.8%) and positive predictive value (94.4%). Hence, HLA-B^*^57 is likely to be an important predictor for anti-TB and ARV drugs co-treatment induced liver injury. However, there could be additional yet unidentified genetic markers and non-genetic risk factors involved in the pathogenesis of DILI. The matched case-control design used in this study minimizes effects of potential confounders and may increase power to identify genetic associations. Although this limits us from exploring associations of the matching variables such as sex, body mass index, Karnofsky score, CD4 count, and HIV viral load which were independently and significantly associated with the risk of developing DILI (Yimer et al., 2011, 2014). Association of CD4 cell counts and Karnofsky score as risk factors for DILI were also found in this study, although the others were not significant.

Studies suggest that a particular *HLA-B* allele may exert a protective effect against certain adverse drug reactions as evidenced by lower allele carrier frequencies in cases compared with treatment tolerants. HLA-DQA1^*^01:02 was identified as a protective variant for anti-TB drugs induced hepatotoxicity (Sharma et al., 2002). HLA-B^*^40:01 and HLA-B^*^07:02 were also identified as protective variant alleles for carbamazepine-induced severe cutaneous adverse reactions (Alfirevic et al., 2006; Hung et al., 2006). In our study, statistically significant lower allele carrier rate of HLA-B^*^41 was noted in the DILI cases compared with the treatment tolerants (6.5 vs. 21.7%), but this effect did not reach statistical significance after correcting for multiple comparisons. Further, analysis is required to clarify the role of HLA-B^*^41 in the prevention of development of anti-TB and ARV drugs co-treatment induced liver injury.

Our result indicated a positive association of HLA-B^*^57 allele with mild-to-moderate liver injury and the HLA-B^*^14 allele with severe liver injury. On the other hand, HLA-B^*^41 allele was negatively associated with mild-to-moderate liver injury. Accordingly, the association of *HLA-B* alleles with anti-TB and ARV drugs co-treatment induced liver injury may seem to depend on the severity of liver injury. The HLA-B^*^57 allele may be critical for the initiation of the immune response to cause DILI and the HLA-B^*^14 allele for the progression to severe degree of liver injury. On the other hand, the HLA-B^*^41 allele seems to play a role in the prevention of development of mild-to-moderate liver injury due to anti-TB and ARV drugs co-treatment. These findings warrant further investigation in a larger DILI case samples for each severity grade of liver injury.

DILI can be hepatocellular (predominant rise in ALT), cholestatic (predominant rise in ALP), or mixed type liver injury (Hussaini and Farrington, 2014). Recently, we conducted a prospective observational study to evaluate the incidence, type, severity, and predisposing risk factors for DILI in a large well-defined TB and/or HIV patient cohort receiving either anti-TB drugs alone, ARV drugs alone or concomitant anti-TB and ARV therapy (Yimer et al., 2014). We found rates of hepatocellular DILI being highest among patients treated with anti-TB drugs alone than patients treated with ARV drugs alone or co-treated with anti-TB drugs. On the other hand, the rates of cholestatic DILI was highest among patients treated with efavirenz based-ARV drugs alone than patients treated with anti-TB drugs alone or with ARV drugs (Yimer et al., 2014). DILI due to anti-TB drugs in TB mono-infected patients is known to be more of hepatocellular type. In the present study, most of the TB-HIV co-infected patients treated with concomitant anti-TB and efavirenz based-ARV drugs developed cholestatic DILI cases. Apparently, there is a significant contribution from efavirenz based-ARV drugs toward developing cholestatic type of DILI. Indeed, the significant association of HLA-B^*^57 variant allele with cholestatic type DILI identified in the present study might reflect for ARV-drugs induced hepatotoxicity. However, the role of HLA variant alleles for predisposition to anti-TB DILI cannot be ruled out (Sharma et al., 2002; Chen et al., 2015). Further, studies are necessary to investigate the association of HLA allele carrier status with anti-TB drugs alone as well as ARV drugs alone-induced liver injury.

Major histocompatibility complex (MHC) class I and class II-mediated immunological reactions are implicated in DILI, particularly in the cholestatic type that involves damage to the biliary system (Andrade et al., 2004; Daly, 2010). In line with this, carrier status of HLA-B^*^57 was significantly higher in patients who presented with the cholestatic type of DILI (44.8%) and mixed type (26.7%) compared with those who tolerated the treatment (2.2%). None of the patients who developed hepatocellular DILI were carriers of HLA-B^*^57 variant allele.

HLA-B^*^57 allele is associated with long-term non-progressive chronic HIV-1 infection by restricting cytotoxic T-lymphocyte response (Goulder et al., 2000). HLA-B^*^57:01 and B^*^57:03 are the most prevalent HLA-B^*^57 subtypes in Caucasian and African populations, respectively (Pelak et al., 2010; Apps et al., 2013). The HLA-B^*^57:01 and B^*^57:03 alleles are protective against HIV disease progression, and appear to present identical Gag epitopes (Payne et al., 2014). HLA-B^*^57:01, B^*^57:02, and B^*^57:03 share more than 90% sequence homology and as such have peptide-binding repertoires which substantially overlap (Illing et al., 2012; Ogese et al., 2017). Although in ART naïve patients HLA-B^*^57 (B^*^57:01 in Europe and US, B^*^57:03 in black Africans) confers protective effect against HIV-1 disease progression to AIDS (Costello et al., 1999; Migueles et al., 2000; López-Larrea et al., 2005; Frater et al., 2007), it may exert contradictory effect on treatment outcome when the disease course is altered by ARV therapy (Dold et al., 2015). Previous studies reported the association of HLA-B^*^57 with increased all causes of mortality (Dold et al., 2015) and reduced virological responses during ARV therapy (Kuniholm et al., 2011). HLA-B^*^57 allele is also known to be associated with immune-mediated drug-induced hypersensitivity reactions. Carriers of HLA-B^*^57:01 allele are at higher risk of developing abacavir-induced hypersensitivity reactions (Hetherington et al., 2002), whereas HLA-B^*^57:03 is associated with spondylarthropathies (López-Larrea et al., 2005). Indeed, genetic screening for HLA-B^*^57:01 variant allele has been shown to reduce drug toxicity and subsequently led to a labeled recommendation of routine screening before treatment initiation (Hughes et al., 2008; Mallal et al., 2008).

The frequency and subtypes of HLA-B^*^57 variant alleles display wide inter-ethnic variability globally ranging from 0 to 22.5% (http://www.allelefrequencies.net/). The overall frequency of HLA-B^*^57 in our study population from Ethiopia is 13% which is relatively high. HLA-B^*^57:01 occurs in Asians and Caucasians (up to 5%). The HLA-B^*^57:03 and B^*^57:02 variant alleles commonly occur in black population reaching up to 3 and 7% allele frequencies, respectively. Interestingly HLA-B^*^57:01 was not detected, and it may be rare or absent in Ethiopians similar to other black Africans where the allele frequency is <1%. The overall HLA-B^*^57:03 and B^*^57:02 allele frequencies in our TB/HIV co-infected study population was 7.6 and 5.4%, respectively, although the frequencies in healthy Ethiopians is yet unknown. Interestingly, HLA-B^*^57:03 and B^*^57:02 allele frequencies in DILI cases (15.2 and 9.8%) were significantly higher than the allele frequencies in the treatment-tolerants (0 and 1.1%), respectively.

There were some limitations in this study. First, as DILI is a rare event, it was not easy to get large number of cases (four years were required to collect the DILI cases in this study), which subsequently resulted in a small number of samples for sub-group analysis. The second limitation is that as drug combinations are the current treatment protocols for TB and HIV co-infections, and hence we cannot link the risk variant allele to a specific drug or class of drug(s). Since first line anti-TB and HIV treatment regimen consists of combination therapy, it is not possible to study individual drug-induced liver toxicity in TB and HIV co-infected patients for ethical reasons. However, our study represents an important first step in applying HLA-B typing to identify genetic variants for anti-TB and ARV drugs co-treatment induced liver injury.

Identification of genetic risk factors for anti-TB, and ARV drugs co-treatment induced liver injury is essential for patient safety. The HLA risk alleles predisposing to immune-mediated anti-TB and ARV drugs induced liver toxicity in black African population are not well investigated. A common problem encountered in HLA genotyping is inability to determine the variant alleles accurately using a simple genotyping procedure. This is mainly due to the extensive genetic diversity in HLA gene locus. Accurate allele-level HLA typing using the current methods requires high workload, cost and time. Because of extreme genetic variation of the HLA locus, pharmacogenetic testing for routine clinical practice is increasingly challenging in resource limited settings. However, the recent development of second-generation sequencing methods provides the possibility of sequencing a single DNA strand in isolation. Establishing a straightforward and affordable genotyping method for accurate HLA typing to identify patients at risk of developing drug-induced adverse events may lay the ground for the future application of pharmacogenetic testing in clinical practice for globalized personalized medicine.

In conclusion, HLA-B variant alleles may play important roles in determining the risk and severity of concomitant anti-TB and ARV drugs induced liver toxicity. HLA-B^*^57 variant alleles (HLA-B^*^57:03 and HLA-B^*^57:02) are risk factors to develop anti-TB and ARV drugs co-treatment induced liver injury, mainly of cholestatic type and mild DILI cases. The possible risk association of HLA-B^*^14 allele with severe DILI and the protective association of HLA-B^*^41 require further investigations. Additional studies are necessary to understand the roles of the identified *HLA-B* variant alleles in the pathogenesis of anti-TB and ARV drugs co-treatment induced liver toxicity.

## Author contributions

EA, JK, and EM conceived and designed the study; EA, GY, AH, EM collected the data; EA, JK, and ZP performed the experiment and analyzed the data; EA and ZP wrote this paper. All authors revised/edited the manuscript and approved for submission.

## Funding

The study was financially supported by grants from European and Developing Countries Clinical Trials Partnership (NL) (CG_TA.05.40204_005) and from Swedish Research Council (Vetenskapsrådet, Grant number: 2015-03295). This work was also supported partly by the NIH/Fogarty International Center Global Infectious Diseases grant D43TW009127.

### Conflict of interest statement

The authors declare that the research was conducted in the absence of any commercial or financial relationships that could be construed as a potential conflict of interest.

## Footnotes

^1^World Health Organization. Ethiopia. HIV/AIDS. Available online at: http://www.afro.who.int/en/ethiopia/country-programmes/topics/4480-hivaids.html (Accessed January 29, 2017).
