# Supplementary material for: HLA-B*57 Allele Is Associated with Concomitant Anti-tuberculosis and Antiretroviral Drugs Induced Liver Toxicity in Ethiopians
Source: Front Pharmacol. 2017 Feb 27;8:90. doi: 10.3389/fphar.2017.00090 (PMC5326775; doi:10.3389/fphar.2017.00090)
Supplement: Supplementary file 1 [file Table1.PDF]

Supplementary Table 1: HLA-B low resolution genotyping for the study participants

Patient No.	Sex	Age (yrs)	BMI (kg/m^2)	Status	Genotype
1	M	32	17.6	Control	B*07 + B*07
2	F	38	19.8	Control	B*41 + B*49
3	F	20	14.5	Control	B*15 + B*73
4	F	26	16.7	Control	B*37 + B*58
5	F	28	19.1	Control	B*41 + B*49
6	F	35	20.2	Control	B*13 + B*13
7	M	36	20.2	Control	B*15 + B*73
8	M	30	17.2	Control	B*15 + B*50
9	F	18	15.4	Case	B*07 + B*14
10	F	45	20.8	Control	B*49 + B*53
11	F	40	24.1	Control	B*14 + B*41
12	F	28	19.4	Control	B*18 + B*44
13	F	25	19.8	Control	B*14 + B*58
14	M	40	20.8	Control	B*41 + B*49
15	M	45	21.0	Case	B*57 + B*57
16	M	55	21.1	Case	B*39 + B*57
17	M	60	18.4	Control	B*44 + B*51
18	M	52	15.2	Control	B*07 + B*44
19	F	36	18.7	Case	B*14 + B*49
20	F	45	17.5	Case	B*57 + B*58
21	F	40	18.8	Control	B*13 + B*44
22	F	30	14.3	Control	B*15 + B*49
23	F	30	16.0	Case	B*53 + B*57
24	M	48	22.0	Control	B*13 + B*41
25	F	35	20.2	Case	B*07 + B*14
26	M	56	19.8	Control	B*08 + B*49
27	M	36	14.6	Control	B*07 + B*41
28	F	30	17.2	Control	B*15 + B*41
29	F	30	15.8	Case	B*44 + B*57
30	M	43	20.2	Case	B*13 + B*14
31	M	32	24.9	Control	B*15 + B*27
32	F	23	22.6	Control	B*39 + B*53
33	F	27	21.9	Control	B*13 + B*53
34	F	27	16.2	Case	B*14 + B*51
35	F	34	21.5	Case	B*57 + B*57

Supplementary Table 1 cont'd

Patient No.	Sex	Age (yrs)	BMI (kg/m^2)	Status	Genotype
36	M	26	18.0	Control	B*39 + B*51
37	F	32	13.6	Case	B*07 + B*15
38	F	24	24.4	Control	B*07 + B*49
39	M	36	23.9	Control	B*07 + B*49
40	M	36	18.1	Control	B*13 + B*49
41	M	21	17.2	Case	B*14 + B*49
42	F	38	16.8	Case	B*49 + B*57
43	F	35	19.4	Control	B*07 + B*15
44	M	35	17.8	Control	B*15 + B*51
45	M	44	16.7	Control	B*13 + B*49
46	F	30	21.8	Control	B*15 + B*50
47	F	30	17.8	Control	B*15 + B*44
48	F	27	17.7	Control	B*07 + B*13
49	F	32	15.1	Control	B*41 + B*57
50	F	30	23.6	Control	B*08 + B*18
51	M	39	15.2	Control	B*07 + B*15
52	F	30	18.4	Control	B*15 + B*41
53	M	47	19.8	Control	B*08 + B*27
54	M	40	15.8	Control	B*07 + B*15
55	F	27	14.3	Control	B*18 + B*18
56	M	40	16.1	Control	B*15 + B*39
57	M	40	17.1	Case	B*07 + B*49
58	M	43	17.3	Control	B*08 + B*13
59	F	28	21.3	Case	B*49 + B*57
60	M	42	19.4	Control	B*14 + B*15
61	F	33	16.7	Control	B*37 + B*51
62	M	33	17.3	Case	B*41 + B*49
63	M	42	32.5	Control	B*15 + B*41
64	F	38	18.4	Case	B*15 + B*39
65	F	37	19.5	Case	B*51 + B*57
66	M	36	14.2	Case	B*07 + B*15
67	M	40	16.2	Case	B*15 + B*39
68	F	20	18.3	Case	B*41 + B*51
69	F	30	19.7	Case	B*14 + B*41
70	M	32	16.8	Case	B*14 + B*14

Supplementary Table 1 cont'd

Patient No.	Sex	Age (yrs)	BMI (kg/m^2)	Status	Genotype
71	M	49	18.6	Case	B*57 + B*58
72	F	46	17.7	Case	B*07 + B*14
73	M	32	19.7	Case	B*49 + B*51
74	M	27	19.0	Case	B*13 + B*15
75	M	41	17.9	Case	B*07 + B*53
76	M	42	16.9	Case	B*15 + B*49
77	F	28	19.6	Case	B*57 + B*58
78	F	30	19.4	Case	B*13 + B*13
79	M	60	16.4	Case	B*13 + B*57
80	M	31	23.7	Case	B*57 + B*57
81	M	26	18.3	Case	B*44 + B*49
82	M	46	20.7	Case	B*51 + B*58
83	M	50	20.2	Case	B*14 + B*49
84	M	38	23.2	Case	B*07 + B*51
85	M	52	20.9	Case	B*07 + B*15
86	F	30	18.4	Case	B*14 + B*15
87	F	30	14.2	Case	B*53 + B*57
88	M	25	16.8	Case	B*57 + B*57
89	F	30	17.1	Case	B*57 + B*57
90	F	39	23.1	Case	B*07 + B*07
91	F	25	16.2	Case	B*57 + B*57
92	F	30	18.7	Case	B*35 + B*53

BMI - Body Mass Index
